# Scarlet Fever in the Lower Animals

**Published:** 1884-10

**Authors:** 


SCARLET FEVER IN THE LOWER ANIMALS.—We invite the attention of our readers to an article by Prof. Walley in the current number of the JOURNAL, in which this distinguished gentleman denies the existence of scarlatina in the lower animals. It will be remembered that Drs. Peters and Stickler, in articles published in former numbers, expressed opposite opinions, believing not only in the existence of the disease referred to in the lower animals, but also its transmissibility to the human subject, and *vice versa.*

The course of study initiated by Mr. Darwin, and since largely developed by other eminent writers, has shown the marvellous inter-dependence of every part of Nature; but no aspect of the question seems to us of so much importance as the one under discussion, as hitherto the researches of scientific men have had reference almost entirely to the application of the theory of evolution to physiological rather than pathological questions.

The diversity of opinion existing on this point in the minds of able writers invests it with special interest, and will awaken still further study and lead to fuller investigation on the subject.

We offer the JOURNAL as a fitting medium for this species of scientific polemics, which will undoubtedly result in a clearer conception of this vital question. C.

